# From Five‐Number Summary to Absolute Heterogeneity: Recent Methodological Advances in Meta‐Analysis With Continuous Outcomes

**DOI:** 10.1111/jebm.70158

**Published:** 2026-06-30

**Authors:** Ke Yang, Jiandong Shi, Jianxin Pan, Jiming Liu, Aiping Lyu, Tiejun Tong

**Affiliations:** ^1^ Department of Statistics and Data Science Beijing University of Technology Beijing China; ^2^ Guangdong Provincial Key Laboratory of Interdisciplinary Research and Application for Data Science and Department of Statistics and Data Science Beijing Normal‐Hong Kong Baptist University Zhuhai China; ^3^ Guangdong Provincial Key Laboratory of Interdisciplinary Research and Application for Data Science Beijing Normal‐Hong Kong Baptist University Zhuhai China; ^4^ Department of Computer Science Hong Kong Baptist University Hong Kong China; ^5^ School of Chinese Medicine Hong Kong Baptist University Hong Kong China; ^6^ Department of Mathematics Hong Kong Baptist University Hong Kong China

**Keywords:** absolute heterogeneity, continuous outcomes, five‐number summary, mean, meta‐analysis, standard deviation

## Abstract

Meta‐analysis with continuous outcomes presents a range of methodological challenges. Among these, two issues have received increasing attention: (i) integrating studies that report only the five‐number summary (such as the median, interquartile range, and range) rather than the sample mean and standard deviation (SD), and (ii) accurately quantifying between‐study heterogeneity. This review first summarizes recent advances in estimating the sample mean and SD from the five‐number summary, covering both normality‐ and non‐normality‐based estimation methods. We also review recently developed skewness tests that help determine when normality‐based estimators are appropriate and present a practical flow chart for integrating studies with five‐number summaries into meta‐analysis. Building on this, we discuss methods for quantifying the heterogeneity, focusing on the widely used relative heterogeneity statistic $I^{2}$ and its limitations, particularly its dependence on study sample sizes. We then review the absolute heterogeneity statistic $I_{A}^{2}$, which quantifies population‐level variation across studies and is invariant to study sample sizes, thus complementing traditional measures. By synthesizing these methodological developments and providing practical guidelines and tools, this review aims to support more rigorous and transparent meta‐analytic practice for continuous outcomes, especially in the presence of nonstandard reporting formats and varying degrees of heterogeneity.

## 1 Introduction

Meta‐analysis has become a fundamental statistical tool for synthesizing evidence across studies and plays a central role in modern evidence‐based practice [1]. The term “meta‐analysis” was first coined by Glass in the context of education research, emphasizing the need for systematic integration to overcome the limitations of individual studies, many of which were small and underpowered [2]. The idea of combining information from multiple studies, however, originated long before the term “meta‐analysis” existed. The earliest identifiable formal attempt to pool empirical findings in medicine likely dates back to Pearson, who aggregated results from multiple studies to examine the preventive effect of serum inoculations against enteric fever [3]. Fisher further introduced statistical methods for combining p values, laying an influential foundation for integrating evidence across independent experiments [4]. A landmark step toward formal meta‐analysis in clinical medicine occurred when Beecher analyzed placebo efficacy across 15 trials, demonstrating the potential of quantitative synthesis [5]. This conceptual framework was first formalized in the seminal monograph by Hedges and Olkin, which established the methodological foundation for meta‐analysis [6]. A second edition, published in 2014, updated and expanded the original work [7]. Another key milestone was the introduction of the random‐effects model (REM), which allows for variation in true effects across studies and provides a principled framework for synthesizing heterogeneous evidence [8]. During the 1990s, the establishment of the Cochrane Collaboration, a global network of healthcare experts producing regularly updated systematic reviews, played a critical role in standardizing and expanding the application of meta‐analytic methods across all areas of health care [9]. Since then, meta‐analysis has become a cornerstone of clinical and epidemiological research, routinely used to strengthen statistical power, improve estimate precision, and draw more generalizable conclusions than any individual study can provide.

A meta‐analysis typically proceeds through several sequential steps as presented in Figure S1. First, researchers identify the research question and define the inclusion and exclusion criteria for eligible studies. Next, relevant studies are located through literature searching and screening. After the eligible studies are determined, data extraction is performed to collect the necessary information from each study, followed by quality assessment of the included studies. The extracted data are then combined in the stage of data synthesis and analysis, where the overall effect is estimated, and the heterogeneity among studies is quantified and evaluated. Additional steps, such as sensitivity analysis, addressing potential bias and outliers, and reporting the results, are then conducted to ensure the robustness and transparency of the findings. In this article, we mainly focus on two components. The first is data extraction and preparation, particularly situations where studies report the five‐number summary instead of the sample mean and standard deviation (SD), which requires appropriate statistical methods to estimate the necessary quantities. The second is the meta‐analysis stage, where we focus on methods for quantifying the heterogeneity among studies.

In meta‐analyses with continuous outcomes, the sample mean and SD are routinely required from individual studies. However, in some studies, due to the potential skewness of data, the whole or part of the five‐number summary rather than the sample mean and SD may be reported, including the minimum and maximum values, the first and third quartiles, and the median. As traditional meta‐analysis can only incorporate the sample mean and SD, five‐number summary data need to be properly handled to prevent information loss or misleading results. This issue first drew attention over two decades ago [10]. The pioneer works proposed estimating the sample mean and SD under the normality assumption, with subsequent research extending these methods to handle non‐normal data. Recently, a flow chart for handling the five‐number summary has also been proposed, by which a skewness test will be performed first to determine whether the methods for normal or non‐normal data should be adopted [11]. Notably, the series of work in the flow chart has been supported by an online calculator [12].

Once study‐level effect sizes and their precision are available, meta‐analysis is typically conducted under either a fixed‐effect model (FEM) or an REM. The choice between these approaches as well as the generalizability of the pooled effect across different study populations depends largely on the extent of heterogeneity across studies. Quantifying the heterogeneity is therefore essential in meta‐analysis. In practice, standardized indices bounded between 0 and 1 are commonly used to summarize the relative contribution of between‐study variability to total variation, providing a unitless measure that is independent of the scale of the effect size and thus comparable across different meta‐analyses. The most widely used heterogeneity statistic is $I^{2}$, which estimates the proportion of the observed variability in study estimates that is attributable to heterogeneity rather than sampling error [13, 14, 15, 16, 17]. However, it depends on study sample sizes and tends to increase as studies become more precise [18]. In fact, Rücker et al. showed that $I^{2}$ approaches 1 as sample sizes grow, even when true differences across studies are clinically negligible [18]. Due to its dependence on study sample sizes, Higgins and López‐López noted that $I^{2}$ should be interpreted as a relative heterogeneity statistic [17]. In response to this limitation, recent research has shifted toward population‐level heterogeneity measures. In particular, Yang et al. proposed an alternative measure that aims to directly measure the heterogeneity between the study populations involved in the meta‐analysis [19]. This measure is independent of study sample sizes and thus serves as an absolute measure of the heterogeneity. For practical use, the authors also introduced the corresponding heterogeneity statistic, $I_{A}^{2}$, tailored to meta‐analysis with continuous outcomes. The property of sample size invariance distinguishes $I_{A}^{2}$ from $I^{2}$ and has motivated its use in applied research. For example, Hong et al. explicitly adopted $I_{A}^{2}$ as their primary heterogeneity statistic, emphasizing its invariance to sample sizes and its ability to capture genuine population‐level heterogeneity, in contrast to the $I^{2}$ statistic [20].

In this review article, we provide a comprehensive overview of methodological considerations for conducting meta‐analysis with continuous outcomes, focusing on two key and closely related issues that arise at different stages of the analysis workflow. Section 2 focuses on methods for handling the five‐number summary in meta‐analysis. Section 3 discusses the core meta‐analytic frameworks, including FEM and REM, and emphasizes the central role of heterogeneity in guiding model choice and evaluating the generalizability of the pooled results. In particular, we review both traditional relative heterogeneity statistic and a recently proposed absolute heterogeneity statistic. Section 4 illustrates the reviewed methods via a real‐world meta‐analysis. Section 5 provides a comprehensive discussion of the reviewed methodology.

## 2 Handling the Five‐Number Summary in Meta‐Analysis

For meta‐analysis with continuous outcomes, the sample mean and SD are the required statistics extracted from each individual study. In some other studies, due to the potential skewness of data, researchers may instead report the whole or part of the five‐number summary $\left\{a,q_{1},m,q_{3},b\right\}$, where a is the minimum value, $q_{1}$ is the first quartile, m is the median, $q_{3}$ is the third quartile, and b is the maximum value. By letting n be the size of data, three commonly encountered scenarios for reporting the five‐number summary include

$$S_{1}=\left\{a,m,b;n\right\},$$

$$\;S_{2}=\left\{q_{1},m,q_{3};n\right\},$$

$$\;S_{3}=\left\{a,q_{1},m,q_{3},b;n\right\}.$$

In the literature, however, few existing meta‐analytical methods can directly incorporate the studies with the sample mean and SD and the studies with the five‐number summary. In early days, the studies reporting only the five‐number summary were often considered to have insufficient data and were therefore excluded from the subsequent meta‐analysis. This practice clearly wastes valuable information, especially when the number of available studies is small.

To tackle this issue, a substantial number of methods have been developed over the past two decades for handling the five‐number summary. At the first stage, researchers proposed methods for estimating the sample mean and SD from the five‐number summary, usually under the normality (or symmetry) assumption of data. During last 10 years, except for the normality‐based methods, research further expanded to handle non‐normal data mainly under specific candidate distributions or by transformation approaches. However, there was not practical guideline about how to adopt these methods for a long time. Recently, a flow chart for handling the five‐number summary in meta‐analysis was proposed. Specifically, the skewness test will be performed first to determine whether the methods for normal or non‐normal data should be adopted. In this section, we comprehensively review the related works to handle the five‐number summary and provide an example to implement the methods in the flow chart.

This section gives a narrative methodological review. To make the literature search process transparent, we conducted a forward‐citation search in the Web of Science Core Collection since 2005, using Hozo et al. [10], Wan et al. [21], Luo et al. [22], and Shi et al. [23] as seed articles, as well as checking the references therein. The search was restricted to the categories “Statistics Probability”, “Mathematical Computational Biology”, “Mathematics Interdisciplinary Applications”, and “Multidisciplinary Sciences”. Papers were considered relevant if they proposed statistical methods, software packages, or online tools for estimating the sample mean and SD (or variance) or for detecting/assessing skewness, from the five‐number summary. Applied papers that merely used existing methods without methodological development were excluded. De Livera et al. in arXiv has also been reviewed [24]. Overall, we have included 17 papers that handle the five‐number summary for meta‐analysis in this section [10, 11, 21, 22, 23, 24, 25, 26, 27, 28, 29, 30, 31, 32, 33, 34, 35].

### 2.1 Estimation for Normal (or Symmetric) Data

#### 2.1.1 Symmetry‐Based Methods

##### 2.1.1.1 Hozo et al. [10]

The study of Hozo et al. [10] is widely recognized as the pioneer work in estimation for the sample mean and SD. On the basis of the assumption of the symmetric distribution for the data under scenario $S_{1}$, the sample mean estimator was proposed as

$$\bar{X}\approx \left\{\begin{array}{cc} \frac{a+2m+b}{4}, & n\leq 25,\\ m, & n>25 \end{array}\right.$$

and the sample SD estimator was proposed as

$$S\approx \left\{\begin{array}{cc} \frac{1}{\sqrt{12}}{\left[{\left(b-a\right)}^{2}+\frac{{\left(a-2m+b\right)}^{2}}{4}\right]}^{\frac{1}{2}}, & n\leq 15,\\ \frac{b-a}{4}, & 15<n\leq 70,\\ \frac{b-a}{6}, & n>70. \end{array}\right.$$

For the sample mean estimator, though it is easy to implement, the stepwise format makes the estimator less precise when the sample size is around 25. Moreover, within the interval of either n≤25 or n>25, the estimator is independent of the sample size, and therefore, the sample size information is totally ignored. The sample SD estimator suffers from the similar issues.

##### 2.1.1.2 Bland [27]

Bland [27] extended Hozo et al. [10] for estimating the sample mean and SD from scenario $S_{1}$ to scenario $S_{3}$. Specifically, the sample mean is estimated as

$$\bar{X}\approx \frac{a+2q_{1}+2m+2q_{3}+b}{8},$$

and the sample SD is estimated as

$$\begin{array}{ccc} S\approx & & \left[\frac{a^{2}+2q_{1}^{2}+2m^{2}+2q_{3}^{2}+b^{2}}{16}+\frac{aq_{1}+q_{1}m+mq_{3}+q_{3}b}{8}\right.\\ & & \;{\left.-\frac{{\left(a+2q_{1}+2m+2q_{3}+b\right)}^{2}}{64}\right]}^{\frac{1}{2}}\;. \end{array}$$

Similarly, these two estimators also suffer from the limitation of completely ignoring the sample size information, resulting in unavoidable information loss in estimation.

#### 2.1.2 Normality‐Based Methods

##### 2.1.2.1 Walter and Yao [33]

Walter and Yao [33] focused on the sample SD estimation when the sample range was available, that is., under scenario $S_{1}$. Note that the expectation of the sample range can be expressed as a function of the sample size n and the true SD under the normality assumption. They proposed to estimate the sample SD as

$$S\approx f\cdot \left(b-a\right),$$

where f is the tabulated conversion function. They referred to the tabulations in the literature to provide the values of f for selected sample sizes in a table. For sample sizes that are not tabulated, the interpolation is required. However, the interpolation may not always be accurate for different sample sizes, making the estimator biased. Moreover, as the estimator lacks an explicit form of f, it is inconvenient for practitioners to use it in routine applications.

##### 2.1.2.2 Wan et al. [21]

Wan et al. [21] proposed to further improve the sample SD estimation under the normality assumption. Under scenario $S_{1}$, their (approximately) unbiased estimator of sample SD was given as

(1) $$S\approx \frac{b-a}{2\Phi^{-1}\left(\frac{n-0.375}{n+0.25}\right)},$$

where $\Phi^{-1}\left(\cdot \right)$ is the inverse cumulative distribution function of the standard normal distribution. With an explicit form that smoothly incorporates the sample size information, estimator (1) is easy to implement in practice and offers improved estimation efficiency compared to the previous methods. For scenario $S_{2}$, their sample SD estimator was given as

(2) $$S\approx \frac{q_{3}-q_{1}}{2\Phi^{-1}\left(\frac{0.75n-0.125}{n+0.25}\right)}.$$

This estimator enjoys the similar properties as that in (1). In addition, these two estimators have been proved to be the (approximate) best linear unbiased estimators (BLUEs) under scenarios $S_{1}$ and $S_{2}$, respectively [26, 34]. These estimators have also shown robustness in simulations when the underlying distribution slightly deviates from a normal distribution. An online calculator is presented to facilitate the practical use [12]. For scenario $S_{3}$, Wan et al. proposed to take the simple average of (1) and (2) [21], yet its statistical properties are less investigated.

##### 2.1.2.3 Luo et al. [22]

Luo et al. [22] studied the optimal sample mean estimation under the normality assumption under all three scenarios. Specifically for scenario $S_{1}$, their optimal sample mean estimator is proposed as follows:

(3) $$\bar{X}\approx w\left(\frac{a+b}{2}\right)+\left(1-w\right)m,$$

where w is the optimal weight assigned to the mid‐range (a+b)/2. They minimize the mean square error of estimator (3) to derive the optimal weight as w=[4Var(m)−2Cov (a+b,m)]/[Var(a+b)+4Var(m)−4Cov(a+b,m)]. Noting that w has a complicated form and is not readily accessible for practical use, the authors further approximated the optimal weight in a simple formula as

$$w\approx \frac{4}{4+n^{0.75}}.$$

It is evident that estimator (3) is a smoothly changing function of n, where the sample size information is well used in the estimation. Similarly, the optimal sample mean estimator under scenario $S_{2}$ is proposed as follows:

(4) $$\bar{X}\approx \left(0.7+\frac{0.39}{n}\right)\left(\frac{q_{1}+q_{3}}{2}\right)+\left(0.3-\frac{0.39}{n}\right)m.$$

It has been proved that the optimal sample mean estimators in Luo et al. [11] are the (approximate) BLUEs for the population mean under the normality assumption [26, 34]. Since then, these sample mean estimators serve as the “rules of thumb” under the respective scenarios, especially for normal data. An online calculator is provided to facilitate the practical use [12].

##### 2.1.2.4 Shi et al. [23]

With a more careful investigation of scenario $S_{3}$, Shi et al. [23] managed to offer the optimal sample SD estimator as

(5) $$S\approx w_{s}\left(\frac{b-a}{2\Phi^{-1}\left(\frac{n-0.375}{n+0.25}\right)}\right)+\left(1-w_{s}\right)\left(\frac{q_{3}-q_{1}}{2\Phi^{-1}\left(\frac{0.75n-0.125}{n+0.25}\right)}\right),$$

where the approximated optimal weight was given as

$$w_{s}\approx \frac{1}{1+0.07n^{0.6}}.$$

Together with the optimal sample mean estimator from Luo et al. [22] as

(6) $$\begin{array}{ccc} \bar{X} & \approx & \left(\frac{2.2}{2.2+n^{0.75}}\right)\left(\frac{a+b}{2}\right)+\left(0.7-\frac{0.72}{n^{0.55}}\right)\left(\frac{q_{1}+q_{3}}{2}\right)\\ & & +\left(0.3+\frac{0.72}{n^{0.55}}-\frac{2.2}{2.2+n^{0.75}}\right)m, \end{array}$$

it has been proved under the normality assumption that the above estimators are the (approximate) BLUEs for the population mean and SD under scenario $S_{3}$, respectively [26, 34]. These estimators have also been incorporated into the online calculator to facilitate the practical use [12].

### 2.2 Estimation for Non‐Normal Data

#### 2.2.1 Distribution‐Fitting Methods

##### 2.2.1.1 Kwon and Reis [29]

Kwon and Reis [29] proposed a simulation‐based estimation for the sample mean and SD by the approximate Bayesian computation. Specifically, by the reported order statistics and the nature of observed variable, an empirical decision about the underlying distribution of data can be made, including, for example, normal distributions, log–normal distributions, exponential distributions, and Weibull distributions. Then the prior distributions for parameters are determined on the basis of the chosen distribution, which are usually the uniform distributions with user‐determined ranges. Then the parameters are generated from the prior distributions. Further based on the generated parameters, one can generate data from the empirically chosen distribution and record the corresponding order statistics. The Euclidean distance between the simulated order statistics and the observed ones is calculated. By repeating the above steps, a set of distances corresponding to generated parameters are recorded. The authors suggested to take an acceptance percentage for the distances, for example, 0.1%, to keep the values of generated parameters with the smallest distances. If one considered the normal distribution as the underlying distribution of data, the estimated sample mean is calculated by the average of the accepted values for the mean parameter, and the estimated sample SD can be similarly calculated. If the chosen distribution is non‐normal, the plug‐in method and the simulation method are suggested. However, the complicated steps and computations hinder the application of the simulation‐based method, though Kwon et al. further proposed the R shiny application (ABCMETAapp) to implement this method [36].

##### 2.2.1.2 McGrath et al. [30]

McGrath et al. [30] proposed two types of estimation methods, where the first one is a distribution‐fitting method, called the quantile estimation method. The quantile estimation method is to first prespecify several candidate parametric families of distributions, including normal distributions, log–normal distributions, gamma distributions, beta distributions, and Weibull distributions. The parameters of each candidate distribution are estimated by minimizing the distance between the true and observed quantiles in the Euclidean distance. The distribution with the smallest distance is treated as the underlying distribution of data, and the sample mean and SD are estimated by the selected distribution. This method is similar to the simulation‐based method, and they share the similar pros and cons [29]. The R package (estmeansd) is available to implement their method.

##### 2.2.1.3 De Livera et al. [24]

De Livera et al. [24] considered a density‐based framework to estimate the sample mean and SD from the five‐number summary. Under scenarios $S_{1}$ and $S_{2}$, they applied the three‐parameter distribution, skew logistic distribution (SLD), to estimate the parameters via percentile matching and then obtain the sample mean and SD estimates. It pointed out that the SLD could reduce to exponential and reverse exponential distributions as special cases and approximate normal and log–normal distributions well. Under scenario $S_{3}$, they used a more complicated four‐parameter distribution, the generalized lambda distribution (GLD), to estimate the parameters therein. This distribution can reduce to uniform, exponential, or logistic distributions as special cases, and it can closely approximate several other distributions, such as log–normal, Cauchy, and normal distributions. Although the assumed distributions are flexible to cover some common distributions, SLD and GLD are not familiar for practitioners, and their application is also complex, despite R code is provided for implementation.

##### 2.2.1.4 Zhang and Li [35]

Zhang and Li [35] proposed a supervised learning framework using neural networks to estimate the sample mean and SD from the five‐number summary. When the whole or part of the five‐number summary is available, they are first fed into some neural networks pretrained on synthetic data for given parametric distributions (e.g., normal and beta distributions), where the sample mean and SD will be provided for each network and the parameters in the given distribution can be uniquely determined. Then the most appropriate distribution is chosen from the given ones according to the distances between the observed order statistics and the expected values under the determined distribution in each network. Once the best‐fitting distribution is identified, the sample mean and SD estimates can be obtained from its corresponding pretrained neural network. The authors have provided an online calculator and a Python package (nn‐st‐meansd) to implement their methods. Note that the key idea in this work is similar to that in Kwon and Reis and the quantile estimation in McGrath et al., whereas the neural network is applied in this field for the first time [29, 30]. However, as a black box method, the neural network does not provide the explicit estimators, which may hinder the implementation of the proposed methods for practical use.

##### 2.2.1.5 Tang et al. [32]

Tang et al. [32] proposed two flexible weighted estimators, the weighted quantile estimation and the minimum distance estimator, to estimate the sample mean and SD from the five‐number summary, addressing key limitations of existing methods, such as normality assumptions, computational burden, and failure to account for the precision of quantiles. The proposed approaches incorporate the inverse‐variance–covariance matrix weight based on the asymptotic distributions of sample quantiles, thereby enhancing both accuracy and precision. For distributions with light tails, extreme values receive lower weights due to their high variability, whereas for heavy‐tailed distributions, these extreme values retain greater influence. The methods are flexible and applicable to different sets of quantiles and various underlying distributions and easily implementable using standard software, including R and SAS. The authors recommend the weighted quantile estimation for routine use due to its intuitive weighting scheme and stable performance.

#### 2.2.2 Transformation‐Based Methods

##### 2.2.2.1 McGrath et al. [30]

In addition to the quantile estimation as introduced in Section 2.2.1, McGrath et al. also proposed a transformation‐based method, that is, Box–Cox method [30]. The Box–Cox method, as its name suggests, assumes that the data after the Box–Cox transformation follow a normal distribution. Taking scenario $S_{1}$ as an example, the Box–Cox transformation parameter is chosen so that the transformed minimum and maximum values are symmetric about the transformed median. Then for transformed data that are normally distributed, estimators (1) and (3) are applied to estimate the sample mean and SD, respectively. Then they are transformed back to the original scale by either the numerical integrals or the Monte Carlo simulations. Although this method can provide estimates for non‐normal distributions, it is less robust in the step of determining the Box–Cox transformation parameter with only several order statistics. Therefore, the misleading estimates can be obtained when the transformation parameter is incorrectly chosen. In addition, there are no explicit formulas for the sample mean and SD estimators, which is not easy for practical use. The authors provided the R package (estmeansd) available to implement their method.

##### 2.2.2.2 Shi et al. [31]

Shi et al. [31] focused on the sample mean and variance estimation on the basis of the log‐normal distributions under all three scenarios [31]. Note that for data from a log–normal distribution, the log‐transformed data follow a normal distribution. Thus, under the log scale, the authors first applied the normality‐based methods in Luo et al. to estimate the sample mean and developed unbiased variance estimators [22]. Then the log‐scale sample means and variances were further transformed back to those under the original scale as final estimates. Both plug‐in and bias‐corrected methods were provided. Although this work explicitly derived the sample mean and variance estimators for the log‐normal distributions, it has not received much attention given that it only considered the log‐normal distributions.

##### 2.2.2.3 Cai et al. [28]

Cai et al. [28], for the first time, developed the maximum likelihood (ML) methods for estimating sample mean and SD from the five‐number summary under all three scenarios [28]. For known distributions (e.g., normal), the ML method directly estimated parameters via the joint density function of order statistics. For unknown distributions, they first applied a Box–Cox transformation to the reported order statistics and estimated the power parameter therein via either ML method or least squares method. Then they estimated normal parameters under the transformed scale and transformed the estimates back to the sample mean and SD under the original scale via Monte Carlo simulation. If the underlying distribution is correctly identified, the ML method performs well as expected. If not, this method may yield misleading sample mean and SD estimates. In addition, there do not exist the closed‐form estimators, which may hinder its application in practice.

#### 2.2.3 BLUEs for Location‐Scale Family

##### 2.2.3.1 Balakrishnan et al. [26]

Balakrishnan et al. [26] provided a unified approach to optimally estimating the sample mean and SD from the five‐number summary under the location‐scale family assumption. The Lagrangian method was applied to determine the optimal coefficients that minimize the variance among all linear unbiased estimators with the reported quantiles. With the normal distribution as a special case, Balakrishnan et al. [26] showed the equivalence between their derived BLUEs and the established ones in Wan et al., Luo et al., and Shi et al., respectively [21, 22, 23]. However, a practical limitation is that the accuracy of this method depends on correctly specifying the underlying distribution (e.g., normal and logistic), requiring a prior assumption. Furthermore, calculating the optimal coefficients requires knowledge of the variance–covariance matrix of order statistics, which can be complex to derive and often necessitates numerical integrals or Monte Carlo simulations.

##### 2.2.3.2 Yang et al. [34]

Independent of Balakrishnan et al., Yang et al. also derived the BLUEs for the sample mean and SD from the five‐number summary under the location‐scale family [26, 34]. Specifically, they applied the generalized least squares approach to derive the BLUEs. Similar to Balakrishnan et al., the primary limitation is also its reliance on the correct specification of the underlying distribution of data [26]. If the distribution is misspecified, the estimators can be less accurate than simpler methods, especially for small to moderate sample sizes. The complexity of the calculations is mitigated by the authors’ provided R package (metaBLUE).

### 2.3 Skewness Tests

#### 2.3.1 Shi et al. [11]

Shi et al. [11] proposed the skewness tests on the basis of the five‐number summary. The null hypothesis is that the data are symmetric under the normality assumption, whereas the alternative hypothesis is that the underlying distribution is skewed away from the normality. Under scenario $S_{1}$, the test statistic is

$$T_{1}=\frac{a+b-2m}{b-a},$$

and the critical region of size 0.05 is

(7) $$\left|T_{1}\right|>\frac{1}{\ln\left(n+9\right)}+\frac{2.5}{n+1}.$$

This indicates that once the test statistic $T_{1}$ falls in the above critical region, the null hypothesis gets rejected, and one can then conclude that the data are significantly skewed. On the contrary, if the skewness test is not rejected, then we follow the common practice that assumes the reported data to be normal. The tests under scenarios $S_{2}$ and $S_{3}$ are also available in Shi et al. [11]. All three tests can be carried out via the online calculator [12].

#### 2.3.2 Balakrishnan et al. [25]

Balakrishnan et al. [25] proposed to estimate the sample skewness from the five‐number summary and considered the similar skewness tests compared with Shi et al. [11]. Under scenario $S_{1}$, their test statistic is exactly the same as $T_{1}$ in Shi et al. [11], whereas they provided the critical values for general size α as ${\left({\left(0.56\alpha +0.01\right)}^{1/4}\ln\left(n\right)+0.53\right)}^{-3/2}$. The tests under the other two scenarios were also provided. However, they did not provide the numerical comparison between their tests and those in Shi et al., which makes practitioners difficult to evaluate the performance of the two works [11].

### 2.4 Flow Chart to Handle the Five‐Number Summary

**FIGURE 1:**
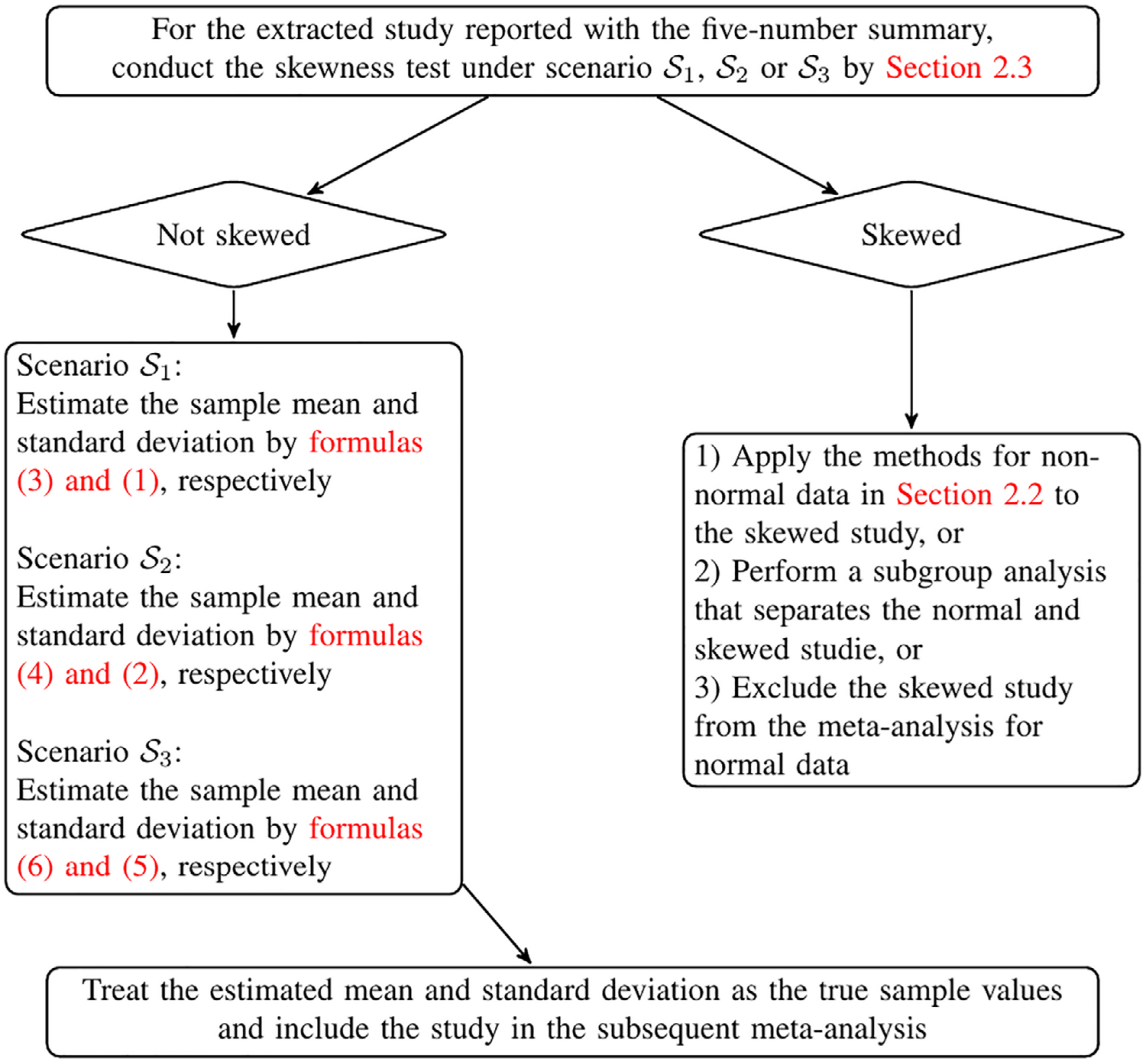
A flow chart to handle the five‐number summary in meta‐analysis with continuous outcomes.

For a long time, there was not practical guideline to handle the five‐number summary, though methods for both normal and non‐normal data were available. Shi et al. provided a flow chart to tackle this issue, and we replotted it in Figure 1 [11]. When a study reported with the five‐number summary is collected, the skewness test under the corresponding scenario is carried out first to detect the potential skewness of data away from normality. If the skewness test is not rejected, one can then apply the normality‐based methods in Section 2.1.2 to estimate the sample mean and SD from the five‐number summary. Specifically, the optimal estimation methods, that is, BLUEs, are recommended in the flow chart [21, 22, 23]. On the other hand, if the study fails to pass the test, three options are provided so that meta‐analysts can determine the most proper one according to their own situations. When there are a considerable number of skewed studies, one can take option (1) to handle them by methods in Section 2.2 to prevent information loss. The reviewed methods for non‐normal data are summarized in Table S1 and most of them can be implemented via R packages or Python package [34, 35, 36, 37, 38]. Additionally, one may take option (2) to perform a subgroup meta‐analysis that separates the normal and skewed studies, if needed. Finally, excluding skewed studies may be considered a last or sensitivity analysis option, particularly when the number of such studies is small; however, this should be done with caution because exclusion may introduce selection bias and reduce generalizability.

To illustrate the implementation of the flow chart, we show a toy example by using the online calculator [12]. Specifically, we consider an example under scenario $S_{1}$ with {a,m,b;n}={3,10,21;50}.

Following the flow chart in Figure 1, we first carry out the skewness test by (7) under scenario $S_{1}$. The test statistic $T_{1}$ is calculated as

$$\frac{3+21-2\times 10}{21-3}\approx 0.222,$$

and the critical value is calculated as

$$\frac{1}{\ln\left(50+9\right)}+\frac{2.5}{50+1}\approx 0.294.$$

Noting that $|T_{1}|\approx 0.222<0.294$, it indicates that the value of the test statistic does not fall in the critical region of size 0.05, and thus the data are not significantly skewed away from normality. Using the online calculator, one can simply input the sample size and the reported order statistics into the corresponding cells under scenario $S_{1}$ (i.e., size = 50, minimum = 3, median = 10, and maximum = 21) and click the “Detect the skewness” button. For this example, the online calculator will report “By Shi et al. (2023), there is no significant evidence to show that the data are skewed. To get the estimates of the sample mean and standard deviation, please click on the ‘Calculate’ button.”

Then given that the data are not significantly skewed away from normality, we can apply the optimal sample mean and SD estimation in Section 2.1.2 according to the flow chart. Specifically, the sample mean estimate is obtained by (3) as

$$\left(\frac{4}{4+50^{0.75}}\right)\cdot \left(\frac{3+21}{2}\right)+\left(1-\frac{4}{4+50^{0.75}}\right)\cdot 10\approx 10.3508$$

and the sample SD estimate is obtained by (1) as

$$\frac{21-3}{2\Phi^{-1}\left(\frac{50-0.375}{50+0.25}\right)}\approx 4.0119.$$

By the online calculator, one can simply click the button “Calculate” to obtain the sample mean and SD estimates directly. A screenshot for implementing the online calculator is shown in Figure S2.

### 2.5 Some Practical Issues

Despite that the reviewed tests and estimation methods are widely used, there are still some practical issues that should draw attention from practitioners. First, the skewness tests described above, rather than serve as definitive tests of the underlying distribution, are better suited for detecting skewness in meta‐analytic studies and guiding the selection of appropriate estimation approaches for subgroup analysis of non‐skewed and skewed data. By serving as an auxiliary tool for pre‐meta‐analysis exploratory analysis of distributional characteristics, it helps researchers tailor the estimation method for the sample mean and SD to the specific features of the five‐number summary, thereby producing more accurate and reliable results. Failure to reject the null hypothesis of symmetry or normality does not necessarily prove that the data are normally distributed. But as the data do not suggest substantial skewness, the normality‐based estimation methods can be practically useful, considering their easy implementation and demonstrated robustness under mildly skewed distributions.

Second, the sample mean and SD estimates from the five‐number summary are not equivalent to the true sample means and SDs. More specifically, they are not equally reliable, which may affect within‐study variances, study weights, pooled estimates, and heterogeneity measures in the subsequent meta‐analysis [39]. Therefore, when feasible, sensitivity analyses should be conducted. For example, meta‐analysts may repeat the analysis after excluding studies with estimated sample means and SDs, compare results obtained using estimation methods for normal and non‐normal data, and examine whether studies reporting five‐number summaries differ systematically from those reporting sample means and SDs directly. If the results are sensitive to the estimation method or to the inclusion of these studies, this should be clearly reported and discussed.

Third, transparent reporting is essential. For studies in which the sample mean and SD are estimated but not directly reported, we recommend reporting the original five‐number summary, the sample size, the applied skewness test, the selected estimation methods, and the estimated sample mean and SD in Table S1. This allows readers to assess the impact of the estimation step and facilitates future reanalysis.

## 3 Quantifying the Heterogeneity in Meta‐Analysis

Once summary statistics, including means and SDs, have been obtained or approximated as discussed in Section 2, they must be transformed into study‐level quantities suitable for synthesis, namely, an observed effect size and a corresponding measure of its precision. Together, these two quantities constitute the core input to meta‐analysis. For continuous outcomes, the study‐specific effect size can take different forms depending on the design. In single‐arm studies, it corresponds to the study‐specific raw mean, whereas in two‐arm studies, commonly used measures include the mean difference (MD) and the standardized mean difference (SMD). For each study i=1,…,k, we collectively denote these effect sizes by $y_{i}$, with their precision represented by the estimated within‐study variance $s_{i}^{2}$.

This section first provides a brief overview of the two commonly used models to synthesize study‐level effect sizes. We then focus on the quantification of between‐study heterogeneity, which is essential for understanding the variability in study effects and for guiding subsequent model‐based inference. This discussion is organized into two complementary perspectives: relative measure of heterogeneity and absolute measure of heterogeneity.

### 3.1 Statistical Models for Meta‐Analysis

Meta‐analytic models specify how the observed study‐level effect sizes relate to the underlying true effects across studies. For the ith study, the observed effect size $y_{i}$ is commonly modeled as

$$\left.y_{i}\right|\theta_{i}∼N\left(\theta_{i},\sigma_{i}^{2}\right),$$

where $\theta_{i}$ denotes the study‐specific true effect, and $\sigma_{i}^{2}$ represents the within‐study variance, which is estimated in practice by the observed within‐study variance $s_{i}^{2}$. Different meta‐analytic models are characterized by the assumptions they make about the collection of true effects ${\left\{\theta_{i}\right\}}_{i=1}^{k}$. These assumptions determine how between‐study heterogeneity is handled, which, in turn, affects the weighting and pooling of study‐level estimates as well as the quantification of uncertainty [15, 40, 41, 42].

#### 3.1.1 Fixed‐Effect Model

FEM is the simplest meta‐analytic model. It assumes that all the included studies share a common true underlying effect [43]. That is, under the homogeneity assumption, we have

$$\theta_{1}=\cdots =\theta_{k}=\theta .$$

Under FEM, the pooled effect provides an estimate of this common effect, with studies weighted according to the precision of their observed effect sizes [44].

The terminology, fixed‐effect, can be easily confused with the fixed‐effects model (with “effects” in the plural), which assumes that study‐specific effects $\theta_{i}$ are fixed but potentially different across studies, and in which the pooled effect typically represents a weighted average of $\theta_{i}$. Although the fixed‐effects model was historically less emphasized due to interpretational ambiguity, it has recently gained practical use [45, 46]. This is especially evident in small‐sample meta‐analysis [42, 47, 48, 49, 50].

#### 3.1.2 Random‐Effects Model

In practice, studies included in a meta‐analysis often differ in their populations, interventions, outcome definitions, and how the studies are conducted. As a result, the true underlying effects are unlikely to be identical across studies, giving rise to what is commonly referred to as between‐study heterogeneity [51]. REM accommodates between‐study heterogeneity by modeling the true study‐specific effects as

$$\theta_{i}∼N\left(\theta ,\tau^{2}\right).$$

where $\tau^{2}$ is the between‐study variance, and the pooled effect estimates the average effect θ across studies [8, 15, 52].

A key component of REM is the estimation of the between‐study variance $\tau^{2}$. The most widely used estimator is the DerSimonian‐Laird (DL) method, which is based on Cochran's Q statistic:

(8) $$Q=\sum_{i=1}^{k}w_{i}{\left(y_{i}-\hat{\theta }\right)}^{2},$$

where $w_{i}=1/s_{i}^{2}$ and $\hat{\theta }=\sum_{i=1}^{k}w_{i}y_{i}/\sum_{i=1}^{k}w_{i}$ [8, 53]. The between‐study variance $\tau^{2}$ is then estimated by

$${\hat{\tau }}_{\mathrm{DL}}^{2}=\max\left\{0,\;\frac{Q-\left(k-1\right)}{\sum_{i=1}^{k}w_{i}-\frac{\sum_{i=1}^{k}w_{i}^{2}}{\sum_{i=1}^{k}w_{i}}}\right\}.$$

Alternative estimators, such as Paule‐Mandel and restricted ML, often provide improved accuracy in small‐sample or highly heterogeneous settings [54, 55, 56, 57, 58, 59, 60, 61, 62]. Exact or nearly exact inference procedures have also been proposed to improve inference for the overall effect under REM in small sample settings [63, 64].

A key distinction between FEM and REM lies in whether between‐study heterogeneity is assumed to be present [14]. Model selection can be guided by testing $\tau^{2}=0$, for example, using Cochran's Q statistic or its refinements [65, 66]. Beyond model selection, heterogeneity is also important for interpreting and generalizing pooled results. It can be quantified in several ways. One common approach is to use the between‐study variance $\tau^{2}$. However, it is known to be specific to a particular effect measure, making comparisons across different meta‐analyses more challenging [8]. Another approach is to use scaled measures defined on a 0–1 scale to describe the degree of heterogeneity. In addition to estimating the average effect θ, prediction intervals are often recommended to reflect the expected range of effects in new settings [16, 41, 67, 68]. In this section, we focus on heterogeneity measures defined on the 0–1 scale.

### 3.2 Relative Measure of Heterogeneity

The heterogeneity characterizes the extent to which study‐specific effects differ and directly affects both the interpretation of pooled results and the choice of meta‐analytic model [69]. An important contribution was made by Higgins and Thompson [13, 14], who proposed the measure as follows:

$$\mathrm{IC}C_{\mathrm{HT}}=\frac{\tau^{2}}{\tau^{2}+\sigma_{y}^{2}},$$

where $\sigma_{y}^{2}$ represents a common or typical within‐study variance [70]. In practice, both variance components are unknown and must be estimated, yielding the widely used $I^{2}$ statistic as

(9) $$I^{2}=\max\left\{0,\;\frac{Q-\left(k-1\right)}{Q}\right\}.$$

Owing to its bounded scale and interpretation as the proportion of total variation due to heterogeneity, $I^{2}$ has been widely adopted in applied meta‐analysis. Comprehensive reviews of its estimation and inference are available [68]. However, $I^{2}$ may be biased in small samples and sensitive to the choice of variance estimators [71, 72], and robust modifications have been proposed [73]. Despite its popularity, the dependence of $\mathrm{IC}C_{\mathrm{HT}}$ on $\sigma_{y}^{2}$ implies that $I^{2}$ is influenced by study sample sizes [15, 18, 74] and should therefore be interpreted as a relative measure of heterogeneity [17]. The use of fixed thresholds (e.g., 50%) to classify heterogeneity or guide model selection lacks theoretical justification.

### 3.3 Absolute Measure of Heterogeneity

Several authors have proposed alternative heterogeneity measures that aim to capture variability on an absolute, population level [75]. Recently, Yang et al. [19] introduced a new heterogeneity measure as follows:

$$\mathrm{IC}C_{\mathrm{MA}}=\frac{\tau^{2}}{\tau^{2}+\sigma_{\mathrm{pop}}^{2}},$$

where $\sigma_{\mathrm{pop}}^{2}$ represents the intrinsic population variance [19].

This measure can be interpreted through a variance decomposition at the individual‐effect level, analogous to the rationale underlying analysis of variance (ANOVA), and intraclass correlation coefficients (ICCs) [4, 76, 77, 78]. In this framework, total variability of individual‐level effects in the study populations arises from within‐population variation $\sigma_{\mathrm{pop}}^{2}$ and between‐population variation $\tau^{2}$. $\mathrm{IC}C_{\mathrm{MA}}$ therefore represents the proportion attributable to differences between study populations.

#### 3.3.1 Properties of $\mathrm{IC}C_{\mathrm{MA}}$

- Boundedness and monotonicity. $\mathrm{IC}C_{\mathrm{MA}}\in \left[0,1\right)$ and increases monotonically with $\tau^{2}$ for fixed $\sigma_{\mathrm{pop}}^{2}$, with values close to 0 indicating little heterogeneity and values close to 1 indicating dominance of between‐study variability.
- Invariance to location and scale transformations. $\mathrm{IC}C_{\mathrm{MA}}$ is invariant to linear transformations of the effect measure (location shifts and rescaling). Therefore, it is comparable across different measurement scales.
- Invariance to the number of studies. $\mathrm{IC}C_{\mathrm{MA}}$ does not depend on the number of studies k, reflecting intrinsic heterogeneity rather than evidence size [79].
- Invariance to study sample sizes. $\mathrm{IC}C_{\mathrm{MA}}$ is defined at the population level and is not affected by study‐specific precision. This contrasts with $\mathrm{IC}C_{\mathrm{HT}}$, which depends on study sample sizes and therefore reflects relative inconsistency rather than absolute heterogeneity [17].

#### 3.3.2 Estimation of $\mathrm{IC}C_{\mathrm{MA}}$ Across Common Effect Size Measures

Yang et al. proposed the $I_{A}^{2}$ statistic as an estimator of ${\mathrm{ICC}}_{\mathrm{MA}}$ for practical use [19]. The computation of $I_{A}^{2}$ depends on the effect size used for continuous outcomes, including the raw mean, MD, and SMD, each corresponding to a different specification of the intrinsic population variance $\sigma_{\mathrm{pop}}^{2}$. In practice, $I_{A}^{2}$ is constructed from study‐level summary statistics $y_{i}$ and $s_{i}^{2}$, with additional adjustment for sample size information.

For raw mean in single‐arm studies, $y_{i}$ is the study mean and $\sigma_{\mathrm{pop}}^{2}$ represents the within‐population variance. The absolute heterogeneity statistic $I_{A}^{2}$ can be calculated directly from Cochran's Q statistic in (8) and the study sample sizes as

(10) $$I_{A}^{2}=\max\left\{0,\;\frac{Q-\left(k-1\right)}{Q+\left(k-1\right)\left(\tilde{n}-1\right)}\right\},$$

where $\tilde{n}=\left(\sum_{i=1}^{k}n_{i}-\sum_{i=1}^{k}n_{i}^{2}/\sum_{i=1}^{k}n_{i}\right)/\left(k-1\right)$ with $n_{i}$ being the sample size of the ith study.

For MD in two‐arm studies, $y_{i}$ is the difference in group means and $\sigma_{\mathrm{pop}}^{2}$ represents the intrinsic variance of individual outcomes within a study arm. The absolute heterogeneity statistic $I_{A}^{2}$ for MD is calculated in the same form as for raw means in (10), with the effective sample size of each study defined as $n_{i}=1/\left(1/n_{Ti}+1/n_{Ci}\right)$, where $n_{Ti}$ and $n_{Ci}$ are the sample sizes of the treatment and control arms, respectively.

SMD is commonly used in two‐arm studies when the outcome scales differ or when the population variances vary substantially across studies [80, 81, 82]. In this setting, MD for each is standardized by the pooled study population SD of its two arms, and SMD is obtained, leading to $\sigma_{\mathrm{pop}}^{2}=1$. Using Hedges’ g as the observed effect size $y_{i}$ [83],F together with its variance $s_{i}^{2}$, the absolute heterogeneity statistic $I_{A}^{2}$ can be computed as

(11) $$I_{A}^{2}=\max\left\{0,\;\frac{Q-\left(k-1\right)}{Q+\left(k-1\right)\left(\tilde{w}-1\right)}\right\},$$

where $\tilde{w}=\left(\sum_{i=1}^{k}w_{i}-\sum_{i=1}^{k}w_{i}^{2}/\sum_{i=1}^{k}w_{i}\right)/\left(k-1\right)$.

**FIGURE 2:**
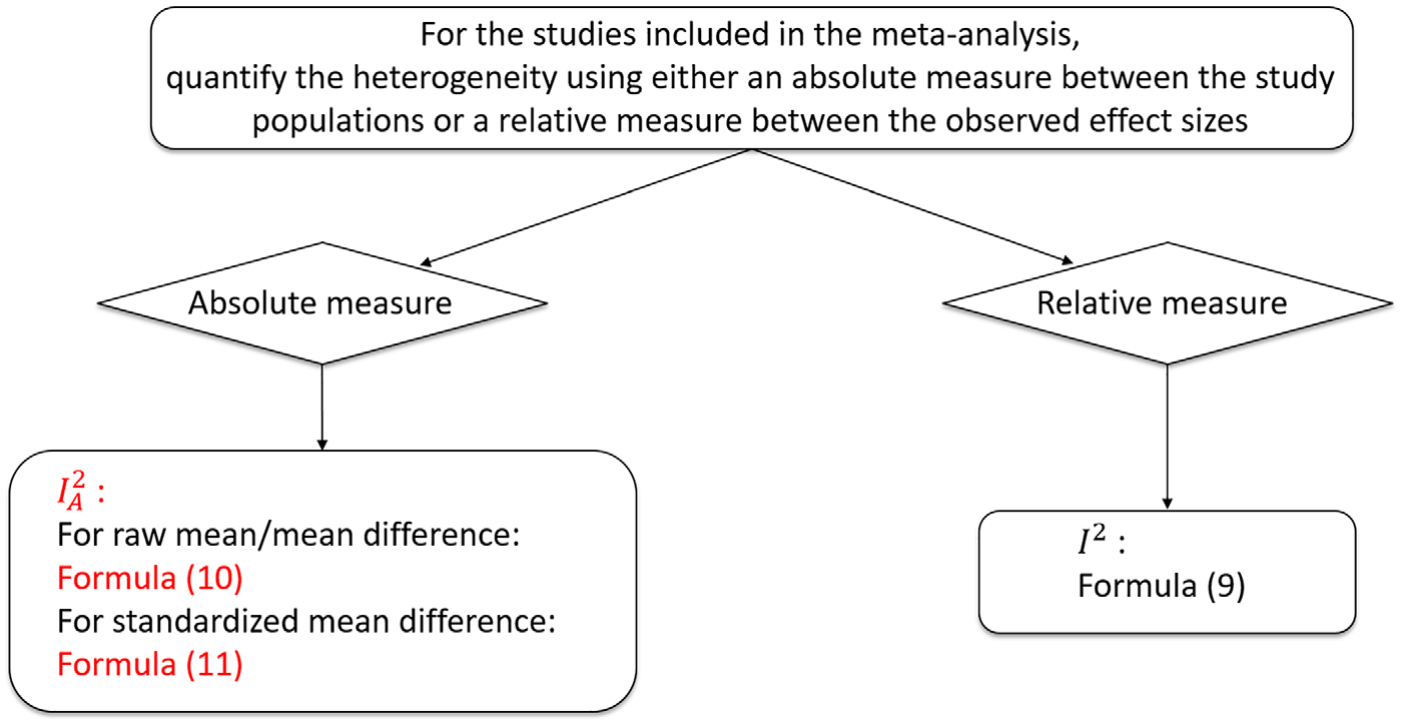
A brief guideline to quantify the heterogeneity in meta‐analysis with continuous outcomes. $I_{A}^{2}$ denotes the absolute heterogeneity statistic, which is invariant to study sample sizes and is defined in formula (10) for raw mean or mean difference and in formula (11) for standardized mean difference. $I^{2}$ denotes the relative heterogeneity statistic, which depends on study sample sizes and is defined in formula (9).

Figure 2 summarizes the proposed absolute heterogeneity statistic $I_{A}^{2}$ alongside the conventional relative heterogeneity statistic $I^{2}$ (Figure 2).

#### 3.3.3 Online Tool for Computing $I_{A}^{2}$

To facilitate practical implementation, the authors have also provided an online calculator for their proposed absolute heterogeneity statistic $I_{A}^{2}$ [84]. The tool is designed to improve accessibility for applied researchers and supports two input modes.

Option 1 applies when summary heterogeneity statistics are available. It requires either Cochran's Q statistic or the conventional $I^{2}$ statistic, with Q recoverable from $I^{2}$ via their standard relationship in (9). Additional inputs are used to recover the intrinsic population variance component, depending on the effect size type: sample sizes for single‐arm studies, arm‐specific sample sizes for MD, and standard errors for SMD (i.e., square roots of within‐study variances).

Option 2 is used when neither Q nor $I^{2}$ is available. In this case, study‐level data are provided directly, from which Cochran's Q is computed internally using standard meta‐analytic procedures, and $I_{A}^{2}$ is subsequently obtained using (10) and (11). Inputs include sample sizes, effect sizes and standard errors for single‐arm studies, and arm‐specific data for two‐arm studies, allowing construction of within‐study variances and weights.

#### 3.3.4 Practical Grading for $\mathrm{IC}C_{\mathrm{MA}}$ and $I_{A}^{2}$

To facilitate practical interpretation, we propose a grading scheme for $\mathrm{IC}C_{\mathrm{MA}}$. We adopt a descriptive classification framework inspired by Landis and Koch [85]. Specifically, the qualitative descriptors (e.g., low, moderate, substantial, severe, and extreme) are defined by adapting the class boundaries originally proposed for Cohen's kappa. Although developed for categorical agreement, this scheme is used here solely as a heuristic tool to provide an interpretable categorization of heterogeneity levels. In practice, $\mathrm{IC}C_{\mathrm{MA}}$ is estimated by $I_{A}^{2}$, and thus the grading criteria are expressed in terms of $I_{A}^{2}$ for direct empirical use. The proposed thresholds are summarized in Table 1.

**TABLE 1:** Grading scheme of $I_{A}^{2}$ for interpreting the extent of the absolute heterogeneity in meta‐analysis.

$I_{A}^{2}$ value	Level of heterogeneity
0.0–0.2	Low
0.2–0.4	Moderate
0.4–0.6	Substantial
0.6–0.8	Severe
0.8–1.0	Extreme

The proposed grading scheme is intended as a descriptive aid for interpreting the magnitude of $I_{A}^{2}$. A value of 0 indicates no heterogeneity. It should not be used as a rigid rule for model selection or decision‐making.

Because $I_{A}^{2}$ provides an absolute, sample size‐invariant summary of heterogeneity, this grading serves as a descriptive tool to contextualize its magnitude. The proposed cutoffs are intended as interpretative guidelines rather than decision rules for choosing between FEM and REM. Model selection should instead consider clinical, methodological, and statistical factors.

## 4 Real‐World Meta‐Analysis

To illustrate the practical use of the methods reviewed in this article, we reanalyzed a real‐world meta‐analysis of Patient Health Questionnaire‐9 (PHQ‐9) scores, which is included in the R package “metamedian” [38]. The dataset consists of 58 primary studies investigating PHQ‐9 scores, where higher values indicate more severe depressive symptoms and scores range from 0 to 27. For each study, the dataset includes the complete set of summary statistics, including the five‐number summary, the sample size, and the sample mean and SD. We considered scenario $S_{1}$ in primary studies, where the minimum, median, maximum, and sample size are available for each study. As several methods for estimating the sample mean and SD require positive and strictly ordered summary statistics, we applied the same type of data adjustment as in McGrath et al. [30].

We first applied the proposed skewness test under scenario $S_{1}$. For each of the 58 studies, the observed value of the test statistic $T_{1}$ was compared with its corresponding critical value at the 0.05 significance level [11]. The test identified 55 studies as significantly skewed, whereas the remaining 3 studies were not detected as skewed. This finding indicates that most studies in this dataset showed evidence of departure from normality/symmetry when only the minimum, median, and maximum were used. More importantly, it demonstrates the practical role of the proposed test: rather than applying a single estimation method to all studies, the test can be used to identify studies for which normality‐based methods may be inappropriate and to guide subsequent subgroup and sensitivity analyses.

**FIGURE 3:**
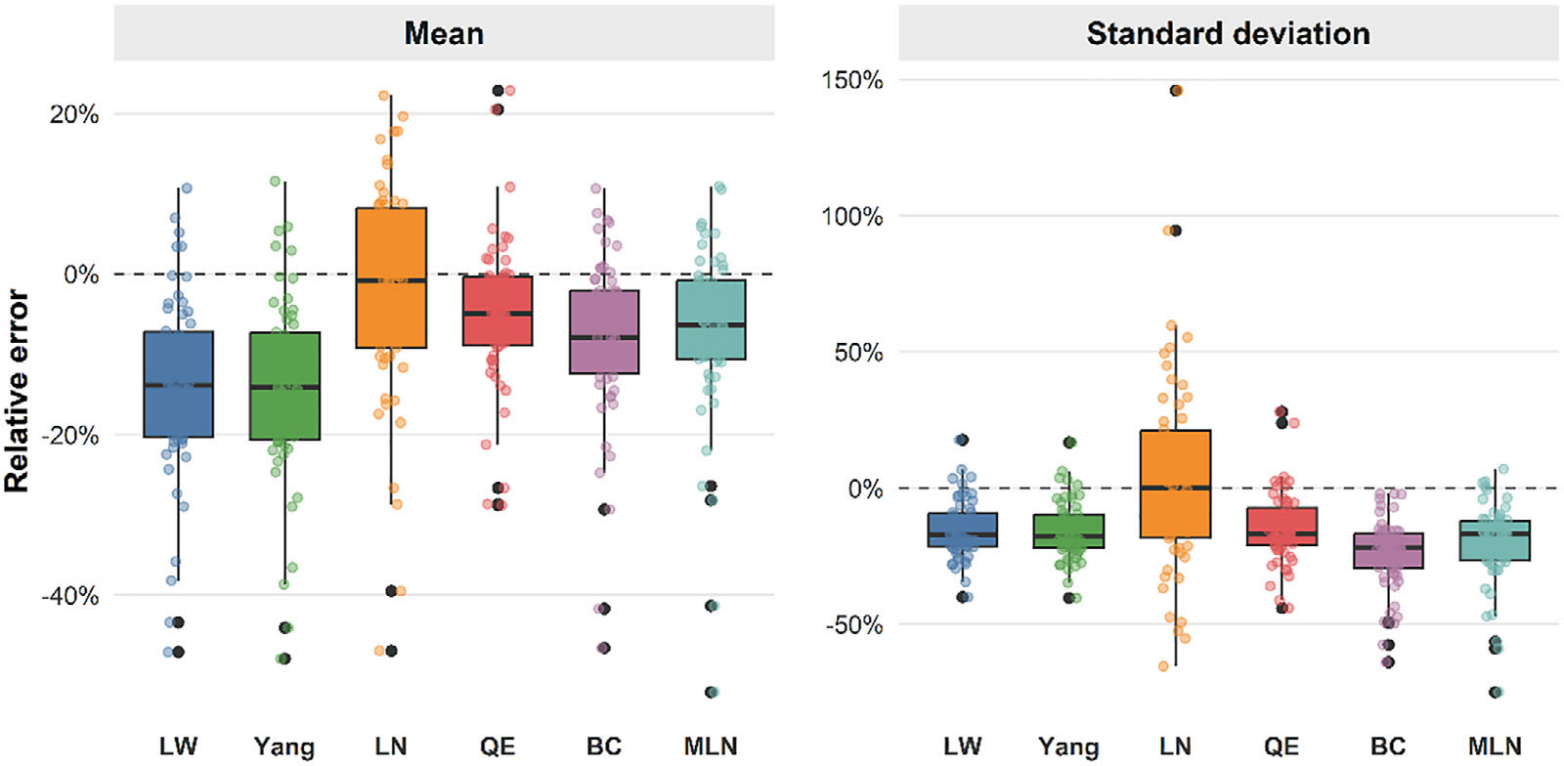
The boxplots of the relative errors for sample mean and standard deviation estimates for different methods. LW, the normality‐based method; Yang, the method for location‐scale family; LN, the method for log‐normal distributions; QE, the quantile estimation; BC, the Box–Cox transformation method; MLN, maximum likelihood estimation.

We then conducted sensitivity analyses using several estimation methods for illustration. For the full set of 58 studies, we compared normality‐based methods of Luo et al. and Wan et al. (LW) [21, 22], the method for location‐scale family in Yang et al. (Yang) [34], and methods for non‐normal data, including the log‐normal method (LN) [31], the quantile estimation method (QE) and the Box–Cox transformation method (BC) of McGrath et al. [30], and the maximum likelihood method (MLN) in Cai et al. [28]. We reported the relative errors of the sample mean and SD estimates for all 58 studies via ChauBoxplot in Figure 3 [86, 87], where the relative errors for the sample mean and SD estimates are calculated by (estimated sample mean − true sample mean)/(true sample mean) and (estimated sample SD − true sample SD)/(true sample SD), respectively (Figure 3). Given that the data from these studies tend to be positively skewed, LW and Yang yielded obviously biased estimates in both sample mean and SD estimates. LN had the smallest median relative error, but it also showed the largest variability across studies. The QE, BC, and MLN methods showed comparatively robust performance with small relative error and variability.

Next, all estimated sample means and SDs were synthesized using an REM fitted by restricted ML. The results are summarized in Table 2, which also reports the corresponding heterogeneity statistics, including $I^{2}$ obtained from formula (9) and $I_{A}^{2}$ obtained from formula (10) (Table 2). When all studies were analyzed together, the LW and Yang methods produced very similar pooled PHQ‐9 scores, 5.76 and 5.73, respectively. The methods for non‐normal data produced larger pooled estimates, ranging from 6.05 to 6.49, where the log–normal method gave the largest estimate. The corresponding heterogeneity results further revealed an interesting pattern. For the conventional $I^{2}$ statistic, all methods except QE method yielded values above 97%, indicating substantial variability among the estimated effect sizes across studies. In contrast, the $I_{A}^{2}$ values were much smaller. Except for the QE method, they ranged from 13.47% to 22.55%. According to the grading scheme in Table 1, these values suggested that the overall heterogeneity across study populations is low or very low.

**TABLE 2:** Meta‐analytical results for all studies, non‐skewed subgroup, and skewed subgroup.

Study	Method	Pooled effect (SE)	${\hat{\tau }}^{2}$ (SE)	$I^{2}$ (%)	$I_{A}^{2}$ (%)
All	LW	5.76 (0.31)	5.43 (1.04)	98.54	18.81
	Yang	5.73 (0.31)	5.32 (1.03)	97.85	13.47
	LN	6.49 (0.35)	6.84 (1.33)	98.84	22.55
	QE	6.22 (0.29)	4.44 (0.91)	94.02	5.12
	BC	6.05 (0.30)	5.09 (1.00)	98.02	14.51
	MLN	6.13 (0.31)	5.25 (1.03)	98.52	18.55
Non‐skewed	LW	12.38 (0.65)	1.03 (1.26)	84.23	4.12
	Yang	12.35 (0.67)	1.00 (1.33)	77.98	2.77
Skewed	LN	6.09 (0.29)	4.31 (0.87)	98.83	21.99
	QE	5.88 (0.23)	2.58 (0.56)	93.40	4.50
	BC	5.70 (0.24)	3.00 (0.62)	97.72	12.49
	MLN	5.77 (0.25)	3.22 (0.67)	98.35	16.52

Abbreviations: LW, the normality‐based method; Yang, the method for location‐scale family; LN, the method for log‐normal distributions; QE, the quantile estimation; BC, the Box–Cox transformation method; MLN, maximum likelihood estimation; SE, standard error; ${\hat{\tau }}^{2}$ represents the between‐study variance estimate.

Guided by the skewness test, we further performed subgroup analyses. For the three studies not classified as skewed, the LW and Yang methods gave pooled PHQ‐9 scores of 12.38 and 12.35, respectively. In contrast, among the 55 skewed studies, the pooled estimates were much lower, ranging from 5.70 to 6.09 across the methods for non‐normal data. This clear difference between the non‐skewed and skewed subgroups highlights the importance of the proposed test. The test not only detects skewness but also reveals that studies with different distributional shapes may contribute differently to the overall pooled result. The heterogeneity results within subgroups show a consistent but more nuanced pattern. Compared with the analysis of all studies, the $I^{2}$ values in both the non‐skewed and skewed subgroups were slightly lower, ranging from 77.98% to 98.83%, yet still indicating substantial variability among the estimated effect sizes. In contrast, the $I_{A}^{2}$ values suggest much weaker overall heterogeneity. For the three studies not classified as skewed, $I_{A}^{2}$ ranged from 2.77% to 4.12%, indicating very low heterogeneity across study populations. Among the 55 skewed studies, except for the LN method, all $I_{A}^{2}$ values were below 20%, also suggesting very low overall heterogeneity across study populations.

Overall, this example demonstrates how the reviewed methods can be applied to the real‐world meta‐analysis where only the median and range are available. The sensitivity analyses show that different estimation methods may lead to different pooled estimates and heterogeneity measures. In addition, the proposed skewness test provides a useful and interpretable way to guide method selection. In the PHQ‐9 example, the test classified most studies as skewed and identified a small non‐skewed subgroup with a markedly different pooled estimate. These findings suggest that detecting skewness before estimation is important, when meta‐analyses rely on studies reported with the five‐number summary.

## 5 Discussion

For continuous outcomes, valid meta‐analytic inference relies not only on the choice of an appropriate pooling model, but also on careful handling of key methodological issues that arise prior to model fitting. In this review, we focus on two such issues that arise at the data‐preprocessing stage and the modeling and inference stage, respectively, and are both essential for obtaining reliable and interpretable results.

The first issue concerns how to properly handle the five‐number summary in meta‐analysis, which is a common data‐preprocessing challenge in practice. We comprehensively reviewed existing works, including the sample mean and SD estimation methods for both normal (or symmetric) and non‐normal data, and the skewness tests. A flow chart by Shi et al. also specifies how to properly apply these methods [11]. Specifically, the skewness tests are first conducted to assess whether the data are skewed away from normality, followed by the selection of suitable estimation methods for the sample mean and SD under different scenarios. This procedure allows practitioners to incorporate eligible studies in a principled manner while acknowledging potential deviations away from normality.

The second issue concerns core meta‐analytic modeling and inference, including the fixed‐effect and random‐effects frameworks, with a particular emphasis on the role of between‐study heterogeneity in guiding model specification and interpreting pooled results. Despite the widespread use of the conventional $I^{2}$ statistic, it measures the relative heterogeneity and is influenced by study sample sizes. We therefore emphasize the role of the absolute heterogeneity statistic, $I_{A}^{2}$, which is invariant to study sample sizes and more directly reflect variability across underlying study populations. Focusing on the absolute rather than relative heterogeneity provides clearer insight into the substantive magnitude of between‐study differences and supports more informed meta‐analytic reasoning. In the context of heterogeneity assessment, $I_{A}^{2}$ provides a promising complementary of absolute heterogeneity to existing methods, including $\tau^{2}$, $I^{2}$, and prediction intervals. It is worth noting that recent literature has also proposed alternative perspectives on the conceptual foundations of meta‐analytic models, emphasizing parameter assumptions rather than classification based solely on statistical models. This viewpoint provides a different interpretation of model choice and generalizability [88].

Taken together, the perspectives summarized in this review highlight the importance of explicitly addressing key issues across different stages of the meta‐analysis workflow, from data preprocessing to model‐based inference. By integrating recent methodological developments with practical considerations, they provide a coherent framework for improving the reliability and interpretability of meta‐analytic practice in continuous outcomes.

## Funding

This work was supported by the National Key Research and Development Program (No. 2024YFA1014100), the National Social Science Fund of China (No. 25CTJ029), the Fundamental Research Funds for Beijing Municipal Universities (312000546325001), the Guangdong Provincial Key Laboratory of IRADS (2022B1212010006), the Guangdong and Hong Kong Universities “1+1+1” Joint Research Collaboration Scheme (2025A0505000010), the General Research Fund of Hong Kong (HKBU12300123), and the Initiation Grant for Faculty Niche Research Areas of Hong Kong Baptist University (RC‐FNRA‐IG/23‐24/SCI/03).

## Conflicts of Interest

The authors declare no conflicts of interest.

## Supporting information




**Supporting File**: jebm70158‐sup‐0001‐SuppMat.docx
