# Supplementary material for: From Five‐Number Summary to Absolute Heterogeneity: Recent Methodological Advances in Meta‐Analysis With Continuous Outcomes
Source: J Evid Based Med. 2026 Jun 30;19(2):e70158. doi: 10.1111/jebm.70158 (PMC13320119; doi:10.1111/jebm.70158)
Supplement: Supplementary file 1 — Supporting File: jebm70158‐sup‐0001‐SuppMat.docx [file JEBM-19-0-s001.docx]

**Supplementary Materials**

**Supplementary Table 1. Summary of Sample mean and standard deviation estimation methods for non-normal data**

| **Publication** | **Assumption** | **Explicit formula** | **Computational burden** | **Robustness** | **Software** |
| --- | --- | --- | --- | --- | --- |
| Kwon and Reis (2015)^1^ | Candidate distributions | No | High | High | R shiny: ABCMETAapp |
| Shi et al. (2020)^2^ | Log-normal distributions | Yes | Low | Low | R package: metamedian |
| McGrath et al. (2020)^3^ | Candidate distributions/ Box-Cox transformation | No | High | High | R packages: estmeansd, metamedian, metaquant |
| Cai et al. (2021)^4^ | Box-Cox transformation | No | High | High | R packages: metafor, metamedian |
| Balakrishnan et al. (2022)^5^ | Location-scale family | No | Moderate | Moderate | Formulas with numerical integrals or simulations |
| Yang et al. (2022)^6^ | Location-scale family | No | Moderate | Moderate | R packages: metaBLUE, metamedian |
| De Livera et al. (2024)^7^ | Skew logistic/generalized lambda distributions | No | High | High | R code;  R package: metaquant |
| Zhang and Li (2026)^8^ | Candidate distributions | No | High | High | Python package: nn-st-meansd |
| Tang et al. (2026)^9^ | Candidate distributions | No | High | High | R code |

**References**

1. Kwon D, Reis I. Simulation-based estimation of mean and standard deviation for meta-analysis via Approximate Bayesian Computation (ABC). *BMC Med Res Methodol*. 2015;15(1):61.

2. Shi J, Tong T, Wang Y, Genton M. Estimating the mean and variance from the five-number summary of a log-normal distribution. *Stat Interface*. 2020;13:519–31.

3. McGrath S, Zhao X, Steele R, Thombs BD, Benedetti A, Collaboration DESD. Estimating the sample mean and standard deviation from commonly reported quantiles in meta-analysis. *Stat Methods Med Res*. 2020;29(9):2520–37.

4. Cai S, Zhou J, Pan J. Estimating the sample mean and standard deviation from order statistics and sample size in meta-analysis. *Stat Methods Med Res*. 2021;30(12):2701–19.

5. Balakrishnan N, Rychtář J, Taylor D, Walter S. Unified approach to optimal estimation of mean and standard deviation from sample summaries. *Stat Methods Med Res*. 2022;31(11):2087–103.

6. Yang X, Hutson A, Wang D. A generalized blue approach for combining location and scale information in a meta-analysis. *J Appl Stat*. 2022;49(15):3846–67.

7. De Livera A, Prendergast L, Kumaranathunga U. A novel density-based approach for estimating unknown means, distribution visualisations and meta-analyses of quantiles. *arXiv*. 2024;2411:10971.

8. Zhang Q, Li Q. Neural network-based estimation of sample mean and standard deviation from some quartiles. *J Syst Sci Complex*. 2026;in press.

9. Tang X, Tong T, Zhang X, Chu H. Minimum distance estimation of mean and standard deviation from reported quantiles. *Res Synth Methods*. 2026:in press.


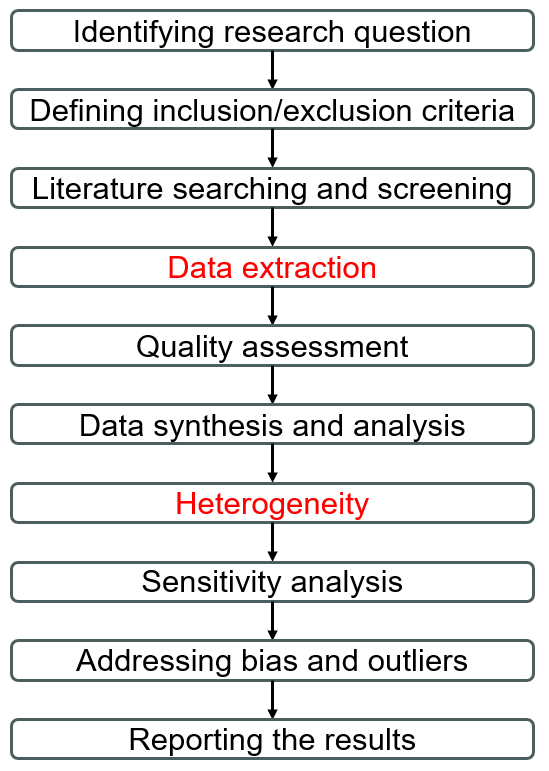


**Supplementary Figure 1. A pipeline for conducting meta-analysis.**

**
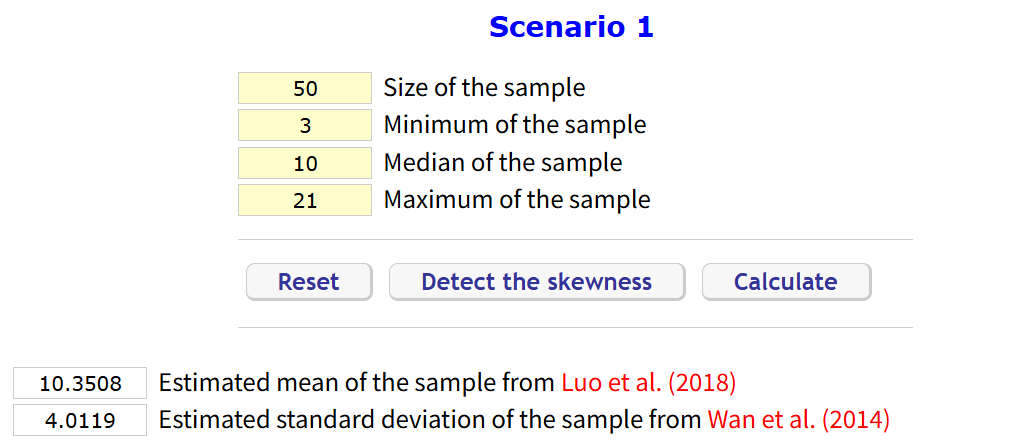
**

**Supplementary Figure 2. An example for implementing the online calculator under scenario** $\boldsymbol{S}_{\mathbf{1}}$**.**
